# Development of Mouse Preantral Follicle after In Vitro Culture in A Medium Containing Melatonin

**DOI:** 10.22074/cellj.2015.499

**Published:** 2015-01-13

**Authors:** Roya Ganji, Mohammad Nabiuni, Roya Faraji

**Affiliations:** 1 Department of Cell and Molecular Biology, Faculty of Biological Sciences, Kharazmi University, Tehran, Iran; 2 Reproductive Health Research Center, Guilan University of Medical Sciences, Guilan, Iran

**Keywords:** Melatonin, Vitrification, Ovarian Follicle

## Abstract

**Objective:**

Improvements in cancer treatment have allowed more young women to survive. However, many cancer patients suffer from ovarian failure. Cryopreservation is one of the solutions for fertility restoration in these patients. The cryopreservation of isolated follicles is a more attractive approach in the long term. Many endocrine and paracrine factors can stimulate the granulosa cells of preantral follicles to proliferate. Melatonin acts as direct free radical scavenger and indirect antioxidant. In this study, we investigated the direct effects of melatonin on follicle development and oocyte maturation by exposing in vitro cultured mouse vitrified-warmed ovarian follicles to melatonin.

**Materials and Methods:**

In an experimental study, preantral follicles with diameter of 150-180 µm were isolated from prepubertal mouse ovaries. Follicles were vitrified and thawed using cryolock method. They were then cultured individually for 7 days in droplets supplemented with 0, 10 and 100 pM melatonin, while ovulation was induced using epidermal growth factor (EGF) and human chorionic gonadotropin (hCG). The survival rate of follicles and nuclear maturation of ovulated oocytes were determined.

**Results:**

At the end of culture, significant increases in follicle survival (p<0.001) and in diameter (p<0.05) were noticed in 10 pM melatonin group compared to control group. In the 100 pM group, survival rate was not affected by melatonin. It was revealed that after induction of ovulation, total number of metaphase II oocytes in treatment groups were not influenced by melatonin (p>0.05).

**Conclusion:**

Culture of mouse vitrified-warmed preantral follicles in a medium supplemented with 10 pM melatonin increased the number of surviving follicles.

## Introduction

Improved cancer treatments have allowed more young women to survive. However, many of these patients suffer from ovarian failure or early menopause that is followed by loss of reproductive function (1, 2). Cryopreservation is a solution for these patients to restore fertility before undergoing chemotherapy or radiation. Furthermore, this technology can be applied for young cancer patients who do not have a male partner (3). However, for particular cancer patients, some options are not suitable (4). Also cryopreservation of mature oocytes has shown limited success (5). Cryopreservation of ovarian tissue enables the storage of the large number of primordial or preantral follicles and preserves the fertility of patients (6, 7). Although autografting of ovarian tissue is a promising method, it has the risk of reimplanting residual cancer cells in patient (8). The cryopreservation of isolated follicles is a more attractive approach in the long term because it would eliminate the risk of reintroducing malignant cells (9) and has many advantages over ovarian tissue cryopreservation. It has been shown that the permeation of cryoprotectant agent (CPA) through the follicular structure is more effective compared to ovarian tissue pieces (10). Also the post thaw assessment of isolated follicles is easier compared to ovarian tissue pieces (11). Culture of preantral follicles is a method used to produce a great number of developmentally competent oocytes; however, developmental competence of these oocytes is lower than those developed *in vivo* (12, 13). It can be related to insufficiency of the *in vitro* environment (14). Many endocrine and paracrine factors can stimulate the granulosa cells of preantral follicles to proliferate. Melatonin is an indolamine that is synthesized from serotonin in the pineal gland and that is secreted in a circadian manner with low levels during the day and high levels during the night (15, 16). In mammals, melatonin activates two distinct melatonin receptors, melatonin receptor type 1A (MT1) and melatonin receptor type 1B (MT2) (15). The expression levels of MT1 and MT2 mRNAs have been detected in human granulosa cells (17). Also melatonin acts as direct free radical scavenger and indirect antioxidant. It detoxifies the highly reactive hydroxyl radicals (4, 18). Excess reactive oxygen species (ROS) can cause oxidative stress, while they can damage molecules and structures of oocyte and granulosa cells in the follicle. ROS must be continuously deactivated to keep only the minimum required amount to maintain normal cell function (19, 20).

Melatonin is a molecule with lipophilic and hydrophilic properties that permits its transfer into many tissues and fluids (21). Many investigations have shown that melatonin can reduce oxidative stress (22, 23). Melatonin is found in human follicular fluid and its concentration is higher than serum at the same time (21, 24). Melatonin concentrations in ovarian follicles increase with follicular growth (25). It is possible that melatonin acts as an antioxidant in the follicle (25). It is shown that melatonin has antiapoptotic effect on different cell types (26).

The aim of this study was to investigate the potential protective effects of melatonin on cultured vitrified preantral follicles, while we investigated the direct effects of melatonin on folliculogenesis and oogenesis by exposing *in vitro* cultured follicles to melatonin.

## Materials and Methods

### Experimental animals

In an experimental study, female and male Naval Medical Research Institute (NMRI) mice were kept in a temperature and light controlled environment (12 hours light: 12 hours dark) and provided with food and water ad libitum. Female offspring, 18-20-day old, were killed by cervical dislocation and ovaries were collected for follicle isolation. All animals were treated in accordance with the guidelines of the Guilan University of Medical Science (GUMS), Rashat, Guilan Province, Iran, for the Care and Use of Laboratory Animals.

### Preantral follicle collection

Ovaries were placed in pre-warmed isolation medium, consisting of α-minimal essential medium (α-MEM) (Invitrogen, USA) supplemented with 10% fetal bovine serum (FBS) (Sigma, Germany), 100 IU/ml penicillin and 100 μg/ml streptomycin (Sigma, Germany). The ovaries were mechanically dissected using 26-gauge needles. Only follicles with diameter of 150-180 μm and central and spherical oocyte, high density of granulosa cells and an intact basal lamina were selected.

### Vitrification procedure

The basal medium for all vitrification and warming solutions was α-MEM medium with 20% FBS. Follicles were equilibrated for 3 minutes in 7.5% ethylene glycol (EG) (Sigma, Germany) + 7.5% dimethyl sulfoxide (DMSO) (Sigma, Germany) followed by 30-40 seconds incubation in vitrification solution containing 15% EG + 15% DMSO with 0.5 M sucrose. After 30-40 seconds, follicles were transferred on cryolock. The cryolocks were plunged into liquid nitrogen and stored for 1-30 days. All steps were performed at room temperature (22-25˚C).

### Warming

For warming, the cryolocks containing the preantral follicles were held at room temperature for 20 seconds. Then the follicles were immersed in 25 μl droplet of 1 M sucrose in the basal medium, kept for 1 minute, and then transferred to 25 μl droplet of 0.5 M sucrose for 3 minutes at room temperature. At the last step, follicles were transferred to droplets of basal medium at 37˚C for 30-40 minutes. Survival rate of vitrified-warmed follicles was assessed under a stereomicroscope (Olympus, Japan). Follicles with naked oocytes or visible spaces between the oocyte and granulosa cell layers or within granulosa cells were considered as damaged. Also dark atretic follicles were considered as damaged. Intact preantral follicles were selected for *in vitro* culture.

### In vitro culture of preantral follicles

Vitrified-warmed preantral follicles were washed three times in culture medium, then were cultured individually in 20 μl droplets of culture medium overlaid with mineral oil (Ovoil, Vitrolife, Sweden) in 60 mm petri dishes (SPL Life Science, Korea). The culture medium consisted of α-MEM supplemented with 5% FBS, Insulin, Transferrin, Selenium (ITS) (Invitrogen, USA), 100 mIU/ml recombinant follicle-stimulating hormone (rFSH) (Gonal-f, Merck-Serono, Germany), 100 IU/ml penicillin and 100 μg/ml streptomycin. Refreshment was performed every other day by removing and replacing 10 μl of medium.

### Experimental groups

To evaluate the effects of melatonin on follicular development and oocyte maturation, we cultured the vitrified-warmed follicles in three groups with the following melatonin (Sigma, USA) concentrations in culture medium: 0 (control), 10 pM and 100 pM.

### In vitro ovulation induction

On day 6 of culture, *in vitro* ovulation was induced by supplementing 1.5 IU/ml recombinant human chorionic gonadotropin (rhCG) (Choriomon, Switzerland) and 5 ng/ml recombinant epidermal growth factor (rEGF) (Sigma, Germany) to the refreshed medium. After 16-18 hours, the ovulated oocytes were denuded by gentle pipetting from cumulus cells for scoring oocyte nuclear maturation state under a stereomicroscope. The diameter of oocytes and follicles was measured at the magnification ×400 using pre-calibrated digital camera under an inverted microscope (LeicaMicrosystems, Inc., USA).

### Statistical analysis

Experiments were repeated independently threefive times. The survival and ovulation rates of follicles and the nuclear maturation of oocytes were analyzed using the χ2 test. Diameter of follicles and oocytes were analyzed with one-way analysis of variance (ANOVA) and data are presented as mean ± SD. Differences at p<0.05 were considered significant. Data analysis was performed using the statistical package for the social sciences (SPSS) (SPSS Inc., Chicago, IL, USA) version 16.

## Results

### Survival of vitrified preantral follicles

**Fig 1:**
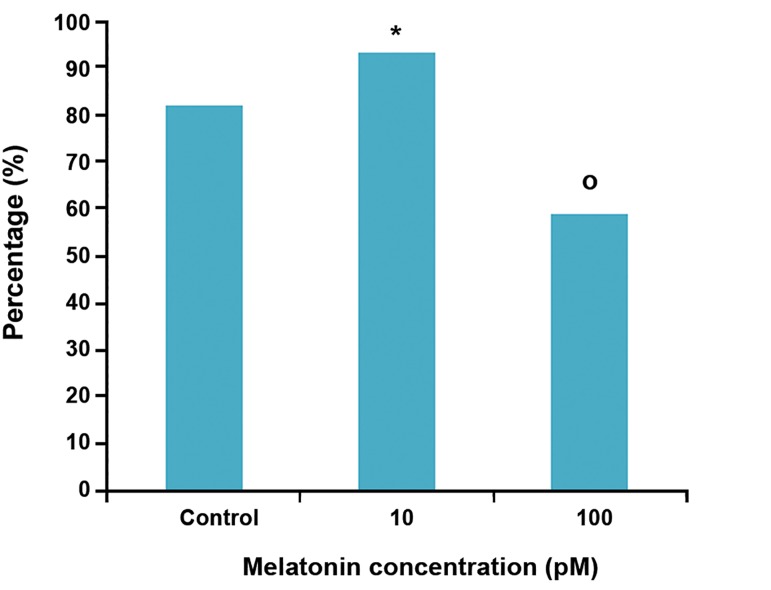
Percentage of surviving follicles in experimental groups on day 7. *; Significant difference with the control group (p<0.05) and o; Significant difference with the control and experimental groups (p<0.05).

Data of survival, ovulation and oocyte maturation belonging to the experimental groups are shown in table 1. On day 7, there was a significant (p<0.05) increase in the number of surviving follicles (93.2%) in 10 pM melatonin group in comparison with control and 100 pM melatonin group. In 100 pM melatonin group, the number of surviving follicles significantly decreased when compared with control and other experimental group (Fig 1).

**Table 1:** Effect of melatonin in culture medium on follicle viability, ovulation and oocyte maturation, cultured *in vitro* for 7 days


Groups	No. of follicles	No. (%) of folliclessurviving	No. (%) of ovulatedoocytes	No. (%) of oocytes with		
				GV	GVBD	MII

**Control**	144	118 (82)	86(73)	19 (17.6)	4 (4.4)	84 (77.9)
**10 pM**	118	110 (93.2)*	86 (77.9)	12 (13.8)	6 (6.9)	73 (79.3)
**100 pM**	131	77 (58.8)*	44 (57.1)*	12 (22.2)	6 (11.1)	36 (66.7)


*; Significant difference with the control group (p<0.05). No; Number, GV; Germinal vesicle, GVBD; Germinal vesicle breakdown and MII; Metaphase II.

### In vitro growth of vitrified preantral follicles

**Fig 2:**
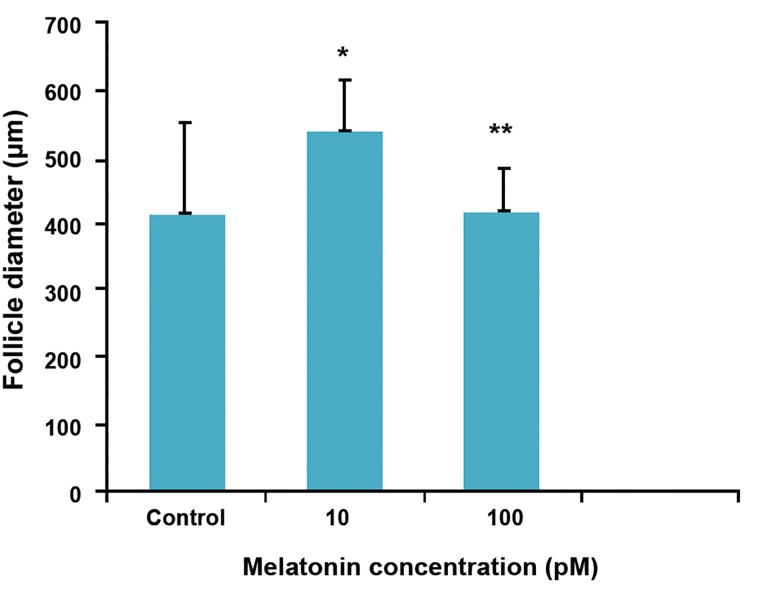
Follicle diameter at the end of culture in control and treatment groups. *; Significant difference with the control group (p<0.05) and **; Significant difference with the 10 pM melatonin (p<0.05).

At the end of culture, the size of follicles in 10 pM melatonin group was larger (546 ± 70 μm) than other groups. The diameter of follicles in this group was significantly (p<0.02) higher than 100 pM melatonin and control groups (426.8 ± 67.8 and 419.9 ± 139.4, respectively, Fig 2).

### In vitro ovulation and oocyte maturation

After *in vitro* ovulation induction, surviving follicles released a mucified cumulus-oocyte complexes (COCs). In 10 pM melatonin and control groups, the number of ovulated oocytes was significantly higher than 100 pM melatonin (p<0.05).

Oocyte maturation rate was not influenced by melatonin. Differences in the number of mature oocytes between all groups were not significant (p>0.05, Figes3, 4).

**Fig 3:**
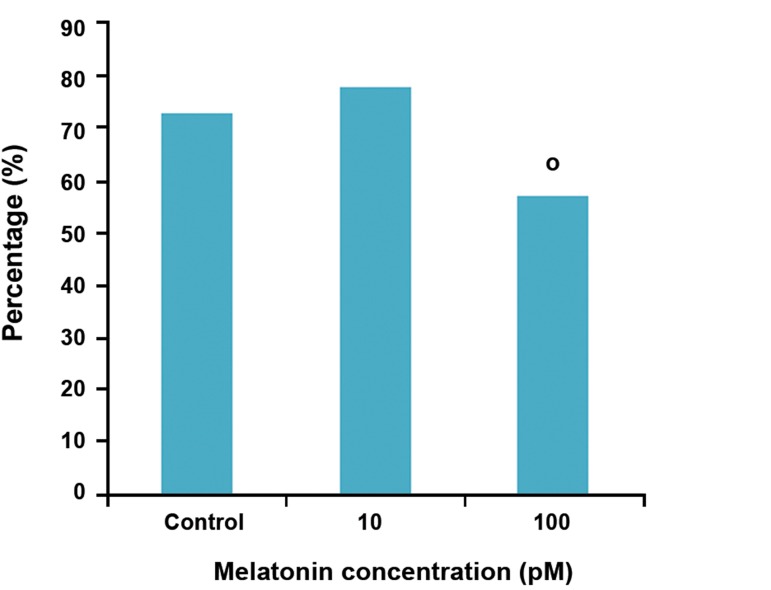
Percentage of cumulus-oocyte complexes (COCs) in experimental groups. o; Significant difference with the control and experimental groups (p<0.05).

**Fig 4:**
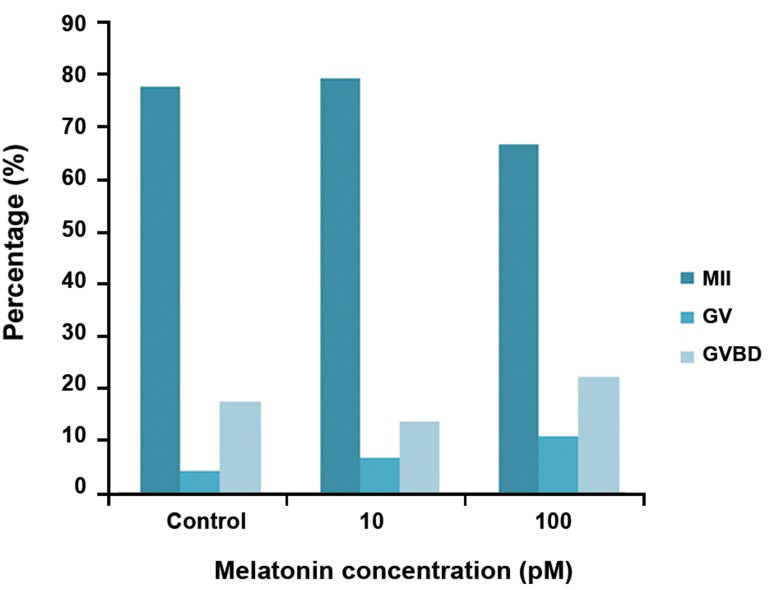
Effect of melatonin on oocyte maturation. M II; Metaphase II oocyte, GVBD; Germinal vesicle break down and GV; Germinal vesicle.

### Oocyte diameter

**Fig 5:**
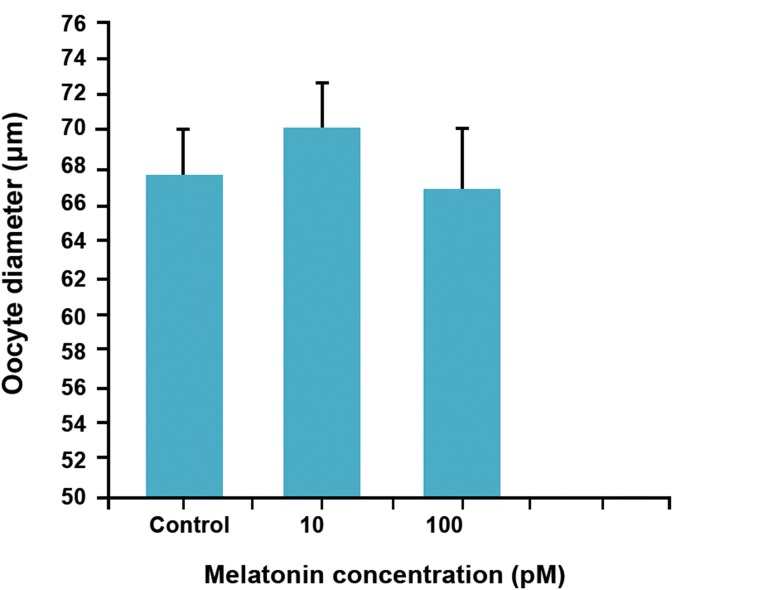
Effect of melatonin on oocyte diameter (ANOVA).

The mean diameter of oocytes in control group was 67.7 ± 2.4 μm that after exposure to melatonin showed no significant increase (p>0.05). The highest diameter of oocyte was observed in 10 pM melatonin goup (70.3 ± 2.6, Fig 5).

**Fig 6:**
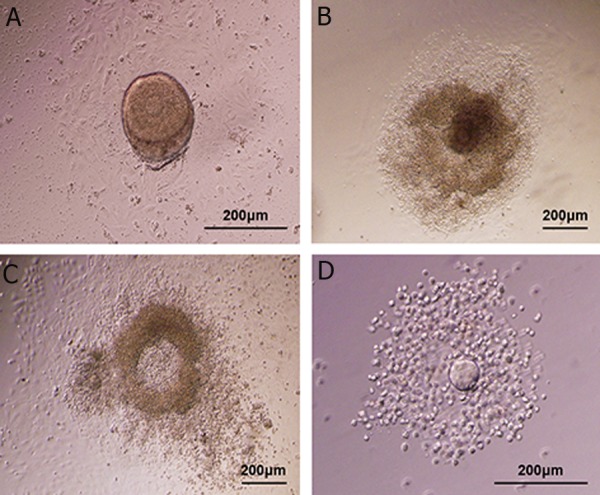
Photographs present *in vitro* follicle development. A. Preantral follicle at day 1 of culture: central germinal vesicle (GV)-stage oocyte with a thin zona, surrounded by granulosa cells. There are some theca cells and a basal membrane attached to the membrane. B. At day 6 of culture, a very diffuse pattern of a growing follicle with a centrally located oocyte is spreading over the monolayer. An antrum-like cavity is formed in some follicles. C. At day 7, a muciﬁed cumulus-oocyte complex ﬂoating free in the culture droplet is produced 16-18 hours post-human chorionic gonadotropin (hCG) stimulation. D. The ovulated oocyte exhibits cumulus expansion and polar body extrusion.

The stages of *in vitro* follicle development are showen in figure 6.

## Discussion

Cryopreservation of preantral follicles is the method for preservation of the large number of primordial and preantral follicles (27). Cryopreservation of oocytes and embryos by vitrification is an efficient method that can be used for both animals and humans (28, 29). Trapphoff et al. (30) reported that vitri.cation by cryotop with DMSO and EG as cryoprotectants is an effective method in preserving preantral follicles. Therefore, we used DMSO and EG as efficient cryoprotectants and cryolock as a derivative of cryotop method for vitrification of preantral follicles. It is demonstrated that the process of freezing and thawing before transplantation of the ovaries generates an excessive metabolic stress in follicles (31). Kim et al. (32) reported that in frozen-thawed ovaries, ascorbic acid reduced apopototic cells. In the other study, during is chemiareperfusion in ovary, vitamin E and tripidil improved survival rate of follicles (33, 34). In the current study, vitrified-warmed follicles showed a high rate of survival when they were exposed to 10 pM melatonin, while exposure of these follicles to higher dose (100 pM) had negative effect on their survival and growth. Adriaens et al. (35) demonstrated that the culture of mouse fresh preantral follicles in the medium containing the highest dose (2 mM) of melatonin significantly decreased follicle survival. It is shown that retarded embryonic development in mouse vitrified 2-cell embryos is accompanied with high levels of ROS that leads to decrease intracellular antioxidant. Therefore, addition of melatonin (10-11- 10-5 M) to the culture medium of vitri.ed mouse 2-cell embryos significantly increases blastocyst formation, the hatched blastocysts and blastocyst cell number. Also the mean apoptotic cell number of blastocysts was reduced. However the highest dose (10-3 M) of melatonin was detrimental (36). ROS produced in the follicle during the ovulation plays a physiological role in this process. Excessive production of ROS induces oxidative stress and damages oocyte and granulosa cells. It seems that the balance between ROS and antioxidants in the follicle is important for the function of oocyte and granulosa cells (25). The required time for culturing follicle to reach preovulatory stage is dependent on the starting size of follicles. Follicles with a diameter between 170 and 200 µm needed 4-5 days to reach a diameter of 400 µm, which is the size of a large antral follicle (37). In this study, the preantral follicles with diameter of 150-180 µm cultured for 6 days. In this time, follicles in 10 pM melatonin group reached the highest diameter (546 ± 70) compared with other groups. It can be partly due to the reduction of apoptosis in granulosa cells in presence of low concentrations of melatonin. It has been reported *in vivo* grown oocytes have greater diameter compared with the oocytes from *in vitro* cultured follicles (38, 39). Papis et al. (40) showed that melatonin improving bovine embryo development are present in a high oxygen environment where free radicals are easily produced. Kang et al. (41) in their study investigated the effects of melatonin on the maturation of porcine oocytes in the medium with or without melatonin supplementation. Inclusion of melatonin (10 ng/ml) during *in vitro* maturation resulted in a greater proportion of mature oocytes, so melatonin significantly decreased the ROS levels in oocytes. Further improvement of culture conditions may help to enhance the developmental competence of the vitri.ed preantral follicles and their oocytes to reach *in vivo* levels.

## Conclusion

These results demonstrate that inclusion of melatonin at a concentration of 10 pM in this vitrified follicle culture system exerts beneficial effects on follicle survival and maturation. Furthermore, 100 pM melatonin signiﬁcantly lowers follicle diameter and oocyte maturation, but there is no significant difference. Therefore, 10 pM melatonin can be used in further studies to investigate its potential protective effect.
